# Perceptions of lecturers and students regarding discriminatory experiences and sexual harassment in academic medicine – results from a faculty-wide quantitative study

**DOI:** 10.1186/s12909-024-05094-x

**Published:** 2024-04-24

**Authors:** Sabine Ludwig, Sabine Jenner, Ralph Berger, Sylvie Tappert, Christine Kurmeyer, Sabine Oertelt-Prigione, Mandy Petzold

**Affiliations:** 1 Institute of Social Medicine, Epidemiology and Health Economics, Charité – Universitätsmedizin Berlin, Luisenstrasse 57, 10117 Berlin, Germany; 2 Institute of Diversity in Medicine, Medical University Innsbruck, Innsbruck, Austria; 3 Equal Opportunities Office, Charité – Universitätsmedizin Berlin, Berlin, Germany; 4 Department for Teaching and Learning, Quality Assurance Section, Charité- Universitätsmedizin Berlin, Berlin, Germany; 5 Department for Teaching and Learning, MediCoach, Charité – Universitätsmedizin Berlin, Berlin, Germany; 6 Department of Primary and Community Care, Radboud University Medical Center, Nijmegen, The Netherlands; 7 Bielefeld University, Bielefeld, Germany

**Keywords:** Discrimination, Harassment, Academic medicine, Students, Lecturers, Higher education, Medical and dental education, Health professions education, Workplace based discrimination

## Abstract

**Background:**

Discrimination and sexual harassment are prevalent in higher education institutions and can affect students, faculty members and employees. Herein the aim was to assess the extent of discriminatory experiences and sexual harassment of students and lecturers at one of the largest teaching hospitals in Europe. We analyze whether there are differences between lecturers and students, different study programs as well as sex/gender differences.

**Methods:**

In an interdisciplinary, iterative process, a semi-standardized questionnaire was developed and sent to *N* = 7095 students (S) of all study programs and *N* = 2528 lecturers (L) at Charité—Universitätsmedizin Berlin, Germany. The study was conducted from November 2018 to February 2019. Besides a broad range of questions on sociodemographic background allowing for diversity sensitive data analysis, they were asked if they had witnessed and/or experienced any form of discrimination or sexual harassment at the medical faculty, if yes, how often, the perceived reasons, situational factors and perpetrators.

**Results:**

The response rate was 14% (*n* = 964) for students and 11% (*n* = 275) for lecturers. A proportion of 49.6% of students (L: 31%) reported that they have witnessed and/or experienced discriminatory behavior. Sexual harassment was witnessed and/or experienced by 23.6% of students (L: 19.2%). Lecturers (85.9%) were identified as the main source of discriminatory behavior by students. Directors/supervisors (47.4%) were stated as the main source of discriminatory behavior by lecturers. As the most frequent perceived reason for discriminatory experiences sex/gender (S: 71%; L: 60.3%) was reported. Women and dental students experienced more discriminatory behavior and sexual harassment.

**Conclusions:**

Discriminatory behavior is experienced by a significant number of students and lecturers, with power structures having a relevant impact. Dental students and women appear to be particularly exposed. Specific institutional measures, such as training programs for lecturers and students are necessary to raise awareness and provide resources. Furthermore, national preventive strategies should be thoroughly implemented to fight discrimination and harassment at the workplace.

**Supplementary Information:**

The online version contains supplementary material available at 10.1186/s12909-024-05094-x.

## Introduction

Discrimination due to e.g., sex/gender, age, parenthood, sexual orientation or nationality and sexual harassment at the workplace and in higher education institutions are important global public health issues. They can affect employees, faculty members and students regardless of their age, sex/gender or position and are also prevalent in academic medicine [1–7]. In the European Union, 40–50% of women have reported some form of sexual harassment at the workplace and a meta-analysis from the United States shows that out of 86,000 respondents, almost two thirds of women experience potentially harassing behaviors and around one fifth sexual harassment at work [7, 8].

Harassment and discrimination include a wide range of behaviors that are considered hostile, abusive, or humiliating by medical trainees and lecturers with deleterious effects on their well‐being and education [1, 4]. They are often unnoticed types of violence that have an impact on the professional identity formation of students as well as on their specialty choices, on the capacity to work as a team, on the affected groups’ or individual’s capacity to reach full potential and thus on general work processes and the study environment [9–11]. This leads to a decline in productivity and to significant losses in scientific outcomes and can have a negative impact on the reputation of the organization and the future health workforce [3, 12–14].

Sexual harassment is a form of gender discrimination that affects women and men in all areas of work and is mostly attributable to hierarchical power relations [15]. According to the International Labour Organization, sexual harassment can occur in one or more of three forms: verbal, nonverbal, or physical [16]. It can range from verbal attacks to unwanted attention to physical attacks, it can include unwanted or unrequested sexualized behavior to being a victim of threatening or aggressive actions [17–19]. It can have a direct impact on health and can lead not only to depression, anxiety disorders, cardiovascular symptoms and burnout, but also chronic back pain, chronic gastrointestinal pain and headaches [20–26].

There are several national and international studies on discrimination and sexual harassment among health care professionals and in academic medicine looking at its impact on faculty members, medical students and students of further health professions, e.g. dental students. Fnais et al. conducted a systematic review and meta-analysis about the risk factors, prevalence and sources of harassment and discrimination among medical trainees [4]. Broad et al. did a survey of a UK medical school population and Bahji and Altomare a systematic review and meta-analysis focusing on resident physicians [1, 27]. Both studies showed that around two thirds of the respondents have experienced discrimination and harassment. Studies on medical education and harassment in academic medicine in Germany also show that sexual harassment and discrimination are prevalent among medical students [28–30]. A study from Brazil demonstrated that more than two thirds of dental students (68%) in three dental schools report discriminatory experiences in the academic environment [31]. Ivanoff et al. showed that female dental students at four dental schools in the U.S., Bulgaria, Brazil, and India report discriminatory experiences and sexual harassment in their study environment [32]. Further studies show the impact of discrimination and harassment of medical students on their health, e.g., resulting in symptoms of posttraumatic stress [33]. Besides medical students, lecturers also experience discrimination and sexual harassment having an impact on the number of publications, their career satisfaction and career advancement [34]. Jenner et al. conducted a study focusing on medical doctors, some also lecturers, at the Charité Medical University Hospital, one of the biggest hospitals in Europe and found that 70% experienced some form of misconduct at their workplace [35, 36]. Further studies from Taiwan and China also show a high prevalence of sexual harassment and discrimination in hospitals and among medical professionals with around 50% having experienced at least one form of workplace violence [37, 38].

To our knowledge, there is no study comparing discriminatory experiences of lecturers and students and no studies on the situations where it occurred. Furthermore, mainly medical students were asked on discriminatory experiences, but there are only few studies of students from other health professions programs, e.g. dental students. To our knowledge, there is no study in Germany assessing discriminatory experiences or sexual harassment among dental students. Therefore, employing a faculty-wide evaluation, we went one step further and included 1) all lecturers at Charité-Universitätsmedizin Berlin, i.e., researchers from all disciplines and faculties including basic sciences, theoretical medical subjects as well as clinical disciplines and 2) students of all study programs at Charité-Universitätsmedizin Berlin, i.e., medicine, dentistry (see Tables 2 and 3) a focus on a broad range of discrimination experiences expanding upon sexual harassment.

Our aim was to analyze, evaluate and compare the extent of discriminatory behavior and sexual harassment at the faculty among students and lecturers, its frequency, the persons or groups of people from whom it emanated, the situations it occurred and the perceived reasons for discrimination. Furthermore, we wanted to analyze if there are differences in the experiences of discrimination and harassment between women and men, students and lecturers with children and without children and between the study programs. The results will help identify and develop preventive measures for students and lecturers to reduce discrimination and sexual harassment at the medical faculty.

## Methods

The survey was conducted from 30.11.2018 to 25.02.2019 at Charité—Universitätsmedizin Berlin using an online questionnaire that was sent to *N* = 7095 students and *N* = 2528 lecturers. Inclusion criteria were being a student or a lecturer of any of the study programs at Charité. Students under the age of 18 were excluded. Due to an individualized TAN-based procedure, there was no possibility of answering the questionnaire twice. The study was approved by the data protection officers of the Charité Campus Mitte. Participants gave formal written consent to the use of their anonymized data for the study.

### Questionnaire development

In an interdisciplinary, iterative process, a semi-standardized questionnaire was developed together with internal and external experts on discriminatory issues, sexual harassment, gender and diversity as well as the Equal Opportunities Officers, faculty members from the quality assurance section of the Department for Teaching and Learning, medical students, and MediCoach (a psychosocial support service for students at Charité). Besides a broad range of questions on sociodemographic background allowing for diversity sensitive data analysis, the students and lecturers were asked about the quality of teaching and learning, resources, infrastructure, study environment, exams, study progress, workload, career and student support. Furthermore, they were asked if they had witnessed and/or experienced any form of discrimination at the faculty, if yes, how often, in which situations, the perceived reasons for discrimination, from whom the discriminatory behavior emanated and in which situations. We also wanted to know if they had witnessed and/or experienced sexual harassment, how often, from whom it emanated and in which forms (see amendment 1).

### Questionnaire administration

An electronic version of the questionnaire was programmed in the evaluation system EvaSys (evasys GmbH, Lüneburg, Germany). A pretest was conducted in October 2018 and further modifications were made based on the feedback. The survey was advertised via the intranet, the student council initiative, social media and posters on campus.

The survey period was extended by five weeks in order to increase the response rate. Students and lecturers were reminded weekly to participate in the survey.

### Statistics

Statistical data analysis was performed using SPSS® Statistics 25.0 (IBM, Böblingen, Germany). Descriptive statistical data analysis includes student and lecturers´ participation percentages and item scores. Significant differences were calculated using the Chi-square test according to Pearson. A *p*-value of < 0.05 was considered statistically significant.

## Results

A total of 964 (14%) students and 275 (11%) lecturers responded to the survey. Table 1 shows the characteristics of the study participants. The sample of students as well as the sample of lecturers was representative of the student population as well as the population of lecturers based on sex/gender and age. The number of enrolled students in the basic undergraduate study programs, the consecutive study programs and further educational study programs in relation to the response rate and percentage of lecturers per study program are shown in Table 2.

**Table 1:** Characteristics of study participants

	**Students**		**Lecturers**	
	n	%	n	%
Response Rate	964	14	275	11
**Sex/Gender**				
Female	637	69,2	133	54,3
Male	272	29,5	103	42
Diverse	1	0,1	1	0,4
No answer	11	1,1	8	3,3
Missings	43		30	
**Age (students; years)**				
≤ 19	84	9,1	NA	
20–24	369	39,9		
25–29	249	26,9		
30–34	139	15,0		
35–39	38	4,1		
≥ 40	36	3,9		
no answer	9	0,9		
Missings	40			
**Age (lecturers; years)**				
< 30	NA		38	15,1
30–39			94	37,3
40–49			72	28,6
50–60			44	17,5
> 60			2	0,8
no answer			2	0,7
Missings			23	
**Having Children**				
Yes	113	12,3	141	55,3
No	796	86,5	100	36,4
No answer	11	1,1	14	5,1
Missings	44		20

**Table 2:** Number of students enrolled per study program in relation to the response rate and percentage of lecturers per study program

	**Students who responded to the survey**	**Total Enrollment number**	**Proportion of lecturers giving courses in the individual study programs**^**c**^
**Basic Undergraduate Study Programs**	**80,5%**	5303	100,%
Medicine	73,0%	4658	100,%
Dentistry	7,5%	645	7,4%
**Consecutive Study Programs**^a^	**16,4%**	542	14,0%
**Further Educational Study Programs**^b^	**3,1%**	327	13,6%

^a^MSc Health Professions Education, MSc Public Health, BA Health Sciences

^b^MSc International Health, MSc Medical Neurosciences, MSc Epidemiology, MSc Molecular Medicine, MSc Public Health

^c^Lecturers give courses in different study programs

## Discriminatory behavior at the faculty

### Students

A proportion of 10.6% of the students indicated that discrimination at the faculty is frequent or very frequent, 70.4% of students reported that it occurs rarely or occasionally and 18.9% that it does not occur. A significant higher proportion (36.2%) of dental students stated that discrimination occurs often or very often, 57.4% that it occurs rarely or occasionally and 6.4% that it do not occur. There were no significant differences between the other study programs.

A proportion of 9.7% of the students have experienced either discriminatory or undervaluing behavior themselves, 20.0% have observed it and 19.8% have both experienced and observed it (Table 3). Of these, significantly more female students have experienced and observed discrimination (*p* < *0.05*) and more students with children (24%; no children: 19%). However, the differences were not significant (*p* = *0.236*). More dental students have experienced and/or observed it (35.5%) compared to students of other study programs (18.6%; *p* = *0,003*).

**Table 3:** Discriminatory or undervaluing behavior and sexual harassment observed and/or experienced by students (S) and lecturers (L) by sex/gender and frequency

Discriminatory or undervaluing behavior observed and/or experienced							Sexual harassment observed and/or experienced				
	**Students (S)** ***n* = 918**			**Lecturers (L)** ***n* = 252**				**Students (S)** ***n* = 920**		**Lecturers (L)** ***n* = 251**	
**n (%)**	**female n (%)**	**male n (%)**	**no answer**	**female n (%)**	**male n (%)**	**no answer**	**n (%)**	**female n (%)**	**male n (%)**	**female n (%)**	**male n (%)**
yes,experienced S: 89 (9,7); L: 29 (11,5)	68 (10,7)	18 (6,7)	2 (18,2)	24 (18,3)	3 (3,0)	1 (12,5)	yes, experienced S: 44 (4,6); L: 19 (7,6)	41 (6,5)	3 (1,1)	14 (10,8)	5 (5)
yes, observed S: 184 (20,0); L: 22 (8,7)	123 (19,4)	60 (22,3)	1 (9,1)	14 (10,7)	8 (8,0)	0 (0)	yes, observed^e^ S: 69 (7,2); L: 5 (2,9)	35 (5,5)	31 (11,5)	4 (3,1)	0 (0)
yes, experienced and observed^a^ S: 182 (19,8); L: 27 (10,7)	148 (23,4)	29 (10,8)	4 (36,4)	17 (13,0)	7 (7,0)	2 (25,0)	yes, experienced and observed S: 34 (3,5); L: 12 (4,8)	32 (5,1)	2 (0,7)	10 (7,8)	1 (1)
No^b^ S: 463 (50,4); L: 174 (69,0)	294 (46,4)	162 (60,2)	4 (36,4)	76 (58,0)	82 (82,9)	5 (62,5)	no^f^ S: 703 (72,9); L: 203 (80,9)	472 (74,6)	217 (80,4)	92 (71,3)	93 (92,1)
n.a							don't know S: 70 (7,3); L: 12 (4,8)	53 (8,4)	17 (6,3)	9 (7)	2 (2)
Missings	46			23			Missings	44		24	
sex/gender difference p-values	**0.000**			**0.011**			sex/gender difference *p*-value	**0.000**		**0.029**	
	**Students (S)** ***n* = 433**			**Lecturers**^d^ **(L) ** ***n* = 71**				**Students (S)** ***n* = 198**		**Lecturers**^h^ **(L) *n* = 45**	
**Frequency n (%)**	**female n (%)**	**male n (%)**	**no answer**	**female n (%)**	**male n (%)**	**no answer**	**Frequency n (%)**	**female n (%)**	**male n (%)**	**female n (%)**	**male n (%)**
once S: 83 (19,2); L: 10 (14,1)	67 (20,1)	14 (14,1)	2 (28,6)	8 (15,7)	1 (6,6)	0 (0)	once S: 51 (25,8); L: 4 (8,9)	36 (24,3)	14 (29,8)	2 (5,6)	2 (28,6)
several times (< 10) S: 296 (68,4); L: 47 (66,2)	216 (66,5)	75 (75,8)	4 (57,1)	34 (66,6)	11 (73,3)	2 (66,6)	several times (< 10)^g^ S: 126 (63,6); L: 31 (68,9)	93 (62,8)	31 (66,0)	27 (75)	2 (28,6)
often (> 10)^c^ S: 54 (12,5); L: 14 (19,7)	42 (12,9)	10 (10,1)	1 (14,3)	9 (17,6)	3 (20,0)	1 (6,7)	often (> 10) S: 21 (10,6); L: 10 (22,2)	19 (12,8)	2 (4,3)	7 (19,4)	3 (42,9)
Missings	531			204			Missings		766	230	
sex/gender difference p-values	0.102			0.810			sex/gender difference p-values	0.643		0.128	

^a^S: diverse: 1 (100); ^b^L: diverse: 1 (100); ^c^L: diverse: 1 (100); ^d^diverse: 0 (0); ^e^S: diverse 1 (100); ^f^L: diverse 1 (100); ^g^S: diverse 1 (100); ^h^diverse 0 (0)

More than two thirds (68.4%) of the students have experienced discriminatory behavior several times (< 10) (Table 3). In terms of frequency, there are no significant differences between male and female students (*p* = *0.102*) or students with and without children (*p* = *0.726*).

### Lecturers

A proportion of 13.6% of lecturers indicated that discrimination is frequent or very frequent. The proportion of lecturers having both experienced and observed discriminatory or disparaging behavior amounts to 10,7% (Table 3). Significantly more female lecturers experienced and/or observed these than their male colleagues (*p* = *0.011*). There were no significant differences between lecturers with or without children (*p* = *0.236*). A proportion of 66.2% had experienced discrimination several times (< 10) (Table 3). There were no significant differences between female and male lecturers (*p* = *0.810*).

No significant differences were found due to age concerning the experience and/or observation or frequency (*p* = *0.940; p* = *0.068*) or between lecturers with or without children (*p* = *0.068*).

## Discriminatory experiences: Perceived reasons, persons and situations

### Students

#### Perceived reasons for discriminatory experiences

**Fig. 1:**
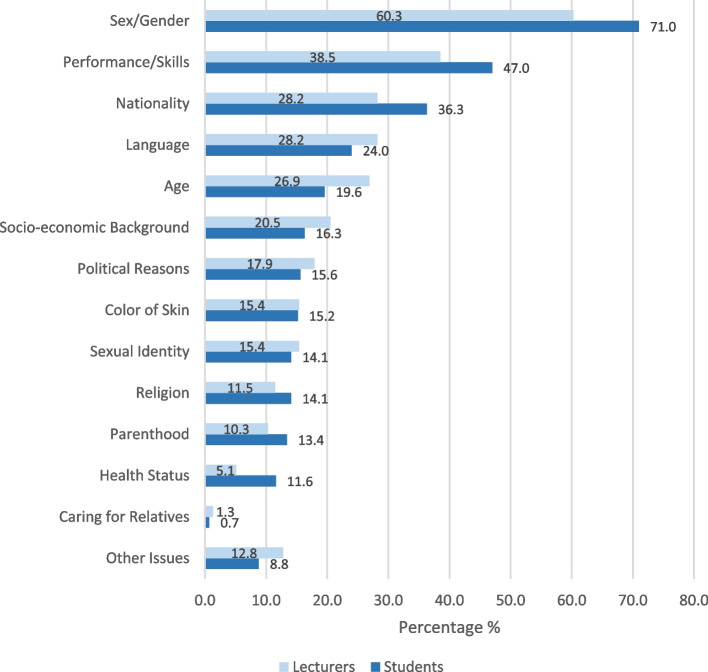
Perceived reasons for discrimination among students (*n* = 455) and lecturers (*n* = 78)

Sex/Gender (71%), performance and skills (47%) and nationality (36.3%) were most frequently cited as perceived reasons for discriminatory experiences (Fig. 1). Compared to students from other study programs, dental students reported significantly more often performance and skills (71.1%) as well as language (28.9%) as perceived reasons for discriminatory experiences.

#### Persons or groups of people from whom discriminatory behavior emanated

**Fig. 2:**
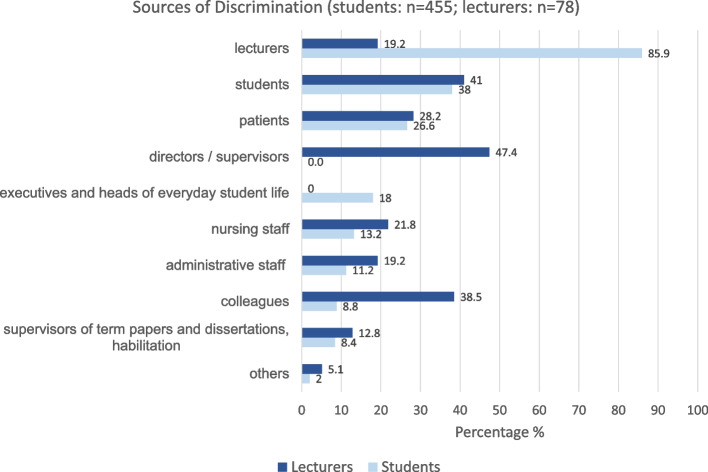
Sources of discrimination (students: *n *= 455; lecturers: *n *= 78)

The persons or groups of people from whom discriminatory behavior emanated were mainly lecturers (85.9%), fellow students (38.0%) and patients (26.6%) (Fig. 2). Female students mentioned colleagues, administrative staff and supervisors of term papers and dissertations more often than male students. The sex/gender differences were significant (*p* = *0.013; p* = *0.021; p* = *0.01*). Dental students named lecturers (*91.1%; p* < *0.001*), executives and heads in everyday student life (48.9%; *p* < *0.001*) significantly more often as persons or groups of people from whom discriminatory behavior originated than students of the other study programs.

#### Situations

Discriminatory behavior was experienced especially in lectures and seminars (72.3%), followed by practical courses (48.1%) and in work situations (28.6%). There are differences between the study programs: Dental students stated that they have experienced discriminatory behavior, particularly during practical courses (59.5%), in work situations (48.6%) and during exams (43.2%). Lectures and seminars were only identified with a share of around one third (35.1%).

### Lecturers

#### Perceived reasons for discriminatory experiences

Lecturers cited sex/gender as the most common reason for discriminatory experiences (60.3%) followed by performance and skills (38.5%) (Fig. 1). Of these, significantly more women (67%, men: 33%; *p* = *0.014*) indicated sex/gender as a reason for discrimination and significantly more men indicated sexual identity (33%; women: 9%; *p* = *0.035*). There were no significant sex/gender differences for the other categories.

#### Persons or groups of people from whom discriminatory behavior emanated

The persons or groups of people from whom discriminatory behavior emanated were mainly directors/supervisors (47.4%), students (41.0%) and colleagues (38.5%) (Fig. 2). Female lecturers named dissertation and habilitation supervisors and directors more often than male lecturers, however the differences were not significant.

Lecturers of dentistry more often named other lecturers and students, each with 55.6%, colleagues (44.4%) and service staff (11.1%). Directors/superiors at work (33%), patients (22.2%) and nursing staff (11.1%) were mentioned less. There were no differences between the other study programs.

#### Situations

Discriminatory behavior was experienced in work situations (66.7%), in lectures and seminars (35.9%), in practical courses (17.9%), on campus (canteen, library) (14.1%) and during exams (10.3%). Lecturers of dentistry more frequently reported lectures and seminars (55.6%), practical courses (44.4%) and discriminatory behavior on campus, but less work situations (33.3%). There were no differences between the other study programs.

## Sexual harassment

### Students

A proportion of 4.8% of students stated that they have experienced some form of sexual harassment during their time at the Charité, e.g. through salacious remarks, unwelcome advances, explicit sexual acts, 7.5% stated that they have observed sexual harassment (Table 3). Female students reported significantly more often that they have experienced or observed forms of sexual harassment (*p* < 0.001). There were no significant differences between students with or without children (*p* = *0.876*) or between the study programs.

Among the students who have witnessed and/or experienced sexual harassment, more than two thirds (63.6%) have witnessed and/or experienced it several times (< 10) (Table 3).

### Lecturers

During their time at the Charité, 7.6% of lecturers of all areas have experienced a form of sexual harassment, lecturers of dentistry report it more often than lecturers of other study programs (11.1%) (Table 3). Significantly more female lecturers experienced and/or observed them (*p* = *0.029*).

Among the lecturers who have witnessed and/or experienced sexual harassment, 8.9% have witnessed and/or experienced it once, 68.9% several times (< 10) and 22.2% often (> 10) (Table 3).

## Sources of sexual harassment

### Students

**Fig. 3:**
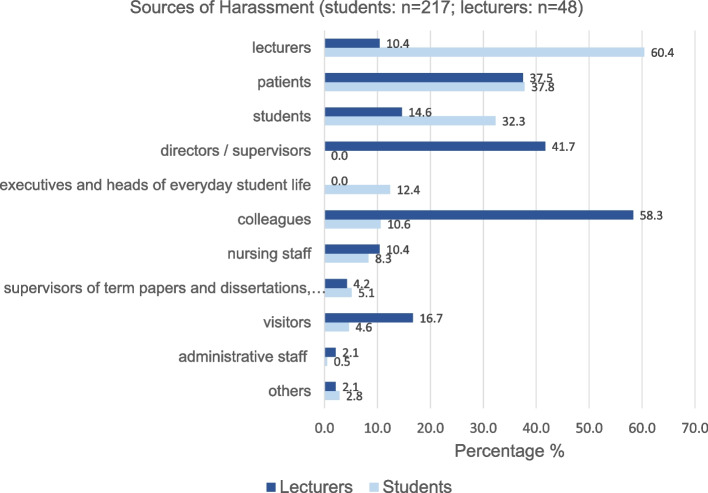
Sources of harassment (students: *n *= 217; lecturers: *n *= 48)

Perpetrators of sexual harassment were mainly lecturers (60.4%), patients (37.8%) and fellow students (32.3%). Harassment by patients, fellow students, executives and heads of everyday student life, colleagues, nursing staff and visitors was more frequently cited by female students. However, the differences were not significant. Compared to students of other study programs, even more dental students indicated lecturers (66.7%) and executives/heads of everyday student life (50%) as main source of sexual harassment (Fig. 3).

### Lecturers

The persons from whom sexual harassment emanated were mainly colleagues (58.3%), directors/supervisors (41.7%) and patients (37.5%). There are no significant sex/gender differences (Fig. 3).

#### Forms of sexual harassment

#####  Students

The most frequent experiences of sexual harassment reported by students were that someone spoke derogatorily of women, men, homosexuals or other sexes (76.5%), or made lewd remarks about their appearance, clothing or sexual allusions or made derogatory remarks (58.1%) or someone made unwanted physical contact, through apparently accidental touching or unnecessary physical proximity (25.3%). There were significant sex/gender differences for some of the items, see Table 4.

**Table 4:** Experiences of sexual harassment by students (*n* = 217) and lecturers (*n* = 48). Multiple answers were possible

**Students (*n* = 217)**				**Lecturers (*n* = 48)**		
**sex/gender differences (*p*-value)**	**Total %**	**Total n**	**Statement**	**Total n**	**Total %**	**sex/gender differences (*p*-value)**
0.754	76.5%	*n* = 166	someone has made sexist, homophobic or other discriminatory sexualized remarks	*n* = 40	83.3%	0.652
**0.000**	5.1%	*n* = 11	someone has sent you or another person derogatory or obscene jokes and sayings or pornographic or nude pictures by telephone, letter, e-mail, SMS or social media	*n* = 4	8.3%	0.879
0.382	58.1%	*n* = 126	someone has made lewd remarks about you or another person, your appearance, your clothing or sexual allusions or derogatory remarks	*n* = 28	58.3%	0.478
0.105	18.9%	*n* = 41	someone has whistled at you or another person unwantedly, stared immorally or gotten undressed with glances	*n* = 17	35.4%	0.741
**0.015**	12.0%	*n* = 26	someone has made intrusive sexual offers or unwanted invitations with sexual intentions	*n* = 11	22.9%	0.662
0.95	0.5%	*n* = 1	someone has promised you advantages if you accept sexual advances, or threatened you with disadvantages if you don't	*n* = 2	4.2%	0.457
**0.029**	25.3%	*n* = 55	someone has made unwanted physical contact, through apparently accidental touching or physically unnecessary proximity	*n* = 17	35.4%	0.543
-	0.5%	*n* = 1	someone has forced you or another person into sexual acts	*n* = 0	0.0%	-

#### Lecturers

As experiences of sexual harassment, the lecturers most frequently cited that someone spoke derogatorily of women, men, homosexuals or other sexes (83%; dental faculty: 25%; *p* = *0.010*), made lewd remarks about their appearance, clothing or sexual allusions or derogatory remarks (58.3%; dentistry: 50%) or that they experienced unwanted physical contact, through apparently accidental touching or unnecessary physical proximity (35.4%; dentistry: 50%). There were no significant sex/gender differences (Table 4).

## Comparison of students and lecturers

Table 5 gives an overview of the main differences reported by students and lecturers. Students report with a proportion of 19,8% (L: 10,7%) that they have experienced and observed discriminatory or undervaluing behavior. More lecturers than students report having experienced and observed sexual harassment (S: 3,5%; L: 4,8%). As perceived reasons for discriminatory experiences, both students and lecturers indicate sex/gender (S: 71%; L: 60%) as the main reason. Students name mainly lecturers as persons or groups of people from whom discriminatory behavior (85.9%) and sexual harassment (60,4%) emanated. Lecturers report that mainly directors/supervisors (41,7%) were the main persons or groups of people from whom discriminatory behavior emanated. Sexual harassment was mainly experienced from colleagues (58,34%) followed by directors/supervisors (41,7%) (Table 5). As main situations where discriminatory or undervaluing behavior occurred students named lectures and seminars (72,3%) and lecturers work situations (66,7%).

**Table 5:** Overview of the main differences between students and lecturers regarding discriminatory or undervaluing behavior and sexual harassment observed and/or experienced

**Discriminatory or undervaluing behavior**		
	**Students (S) *n* = 918, n (%)**	**Lecturers (L) *n* = 252, n (%)**
yes, experienced and observed	182 (19,8)	27 (10,7)
Main Perceived reasons (S: n = 455; L: 78)		
Sex/Gender	**323 (71,0)**	**47 (60,3)**
Performance and Skills	214 (47,0)	30 (38,5)
Nationality	165 (36,3)	22 (28,2)
Sources (S: *n* = 455; L: *n* = 78)		
Lecturers	**391 (85,9)**	15 (19,2)
Students	173 (38,0)	32 (41,0)
Patients	121 (26,6)	22 (28,2)
Directors/Supervisors	38 (8,4)	**37 (47,4)**
Colleagues	40 (8,8)	30 (38,5)
Situations (S: *n* = 455; L: *n* = 78)		
Lectures and seminars	**329 (72,3)**	28 (35,9)
Practical courses	219 (48,1)	14 (17,9)
Work situations	130 (28,6)	**52 (66,7)**
**Sexual harassment**		
	**Students (S; *n* = 920) n (%)**	**Lecturers (L; *n* = 251) n (%)**
yes, experienced and observed	34 (3,5)	12 (4,8)
Sources S: (*n* = 217; L: *n* = 48)		
Lecturers	**131 (60,4)**	5 (10,4)
Students	70 (32,3)	7 (14,6)
Patients	83 (37,8)	18 (37,5)
Directors/Supervisors	n.a	20 (41,7)
Colleagues	23 (10,6)	**28 (58,3)**

## Discussion

The aim of our study was to assess the extent of discriminatory experiences and sexual harassment of students and lecturers at one of the largest teaching hospitals in Europe and analyze whether there are differences between lecturers and students, study programs and women and men. For this an online questionnaire was sent to students of all study programs and all lecturers of the faculty.

Our results show that there is a great extent of discrimination and sexual harassment at the faculty in regard to both lecturers and students. More students than lecturers report that they have experienced or observed discriminatory behavior, however more lecturers have experienced and/or witnessed some form of sexual harassment during their time at the Charité, e.g. through salacious remarks, unwelcome advances, explicit sexual acts. As perceived reasons for discriminatory experiences, both students and lecturers indicate that sex/gender as the main reason followed by performance and skills and nationality. The students indicate that sexual harassment mainly emanated from lecturers, followed by patients and fellow students, the lecturers report that sexual harassment emanated mainly from colleagues, directors/bosses and patients.

The students have experienced discriminatory behavior mainly in lectures, seminars and in practical courses, but less in work situations. Around two thirds of the lecturers have experienced discriminatory behavior mainly in work situations and around one third in lectures and seminars. Female students and faculty experience more discrimination and sexual harassment. Differences between the study programs exits, mainly for dentistry.

A meta-analysis of 51 studies on the prevalence, risk factors, and sources of harassment and discrimination among medical trainees by Fnais et al. showed that around two-third of medical trainees had experienced at least one form of harassment or discrimination during their training [4]. A meta-analysis by Bahji et al. showed similar results [27]. This is also the case for dental students [31]. Karim and Duchcherer reviewed 10 articles [39]. They found that 45–93% of medical residents reported intimidation and harassment in at least one occasion. Verbal abuse was mentioned as the most predominant form of abuse.

In a study by Crutcher et al. 44.7% of family medicine graduates had experienced intimidation, harassment and/or discrimination (IHD) during residency [40].

Broad et al. conducted a survey of a UK medical school population in 2014. Harassment and discrimination are prevalent in this sample. Most participants had experienced or witnessed.

or witnessed at least one type of discrimination or harassment and associated it with gender, ethnicity, sexuality, disability and year group [1]. In our study, the main perceived reasons for discrimination cited by the students were sex/gender, performance and skills and nationality, but also language and skin color.

According to our results, the persons or groups of people from whom discriminatory behavior emanated were mainly lecturers, but also other students, patients and nursing staff. Bahji et al. show that the most common sources of IHD were relatives/friends of patients, nurses, and patients. As they looked mainly at resident physicians, this might be the reason that lecturers and fellow students were cited less as a source of IHD [27]. Crutcher et al. showed that the persons from whom IHD emanated were mainly specialist physicians, followed by hospital nurses, specialty residents, and patients [40].

Our results show that around one third of dental students indicate that discrimination occurs very often or often, compared to around ten percent of other students.

Dental students indicate significantly more often performance and skills as well as language as perceived reason for discrimination. Dental students name lecturers, executives and heads in everyday student life significantly more often as persons or a group of persons from whom discriminatory behavior originated. Garbin et al. evaluated the experiences of sexual harassment in a dental school in Brazil. In their study, patients were cited as the main source of harassment, followed by faculty members; other dental students were cited less frequently [41]. A study by Webster et al. shows that almost 15% of dental students report sexual harassment at least once, female students are more often sexually harassed and students of higher study years more often than first year students [42].

One third of dental students indicate that they have witnessed and/or experienced sexual harassment often, as compared to around ten percent of the other students. The reasons for this might stem from even stricter hierarchies in dental faculties. This is line with findings from international studies [41]. To our knowledge, there are no studies on sexual harassment and discrimination referring only to dental lecturers.

Jenner et al. conducted a study on sexual harassment in a tertiary education facility, at Charité, focusing on the physicians. A proportion of 70% reported some form of misconduct while performing their work [36]. International studies also show high rates of sexual harassment among health care professionals [37, 38, 43–45].

As experiences of sexual harassment, lecturers most frequently cite that someone spoke derogatorily of women, men, homosexuals or other sexes. According to Jenner et al. the most common form of self-reported harassment was verbal harassment; including degrading speech and sexualized speech. The persons from whom sexual harassment emanated were mainly colleagues, directors/supervisors and patients. Jenner et al. also found that colleagues were cited as the main perpetrators [36].

We found that significantly more female students have experienced and observed discriminatory or undervaluing behavior and sexual harassment. National and international studies find the same results [1, 29, 30]. Carr et al. showed that about half of female faculty but few male faculty experienced some form of sexual harassment: Female faculty were more than 2.5 times more likely to perceive gender-based discrimination in the academic environment than male faculty [34].

The results of Jagsi et al. also show that women were more likely than men to report having personally experienced sexual harassment. Age was not a significant factor for students and lecturers in our study [46]. Carr et al. however, found age differences among women with the younger faculty reporting lower rates of discrimination than the older faculty [34].

More students with children have experienced and observed discriminatory behavior at the faculty. Verniers and Vala showed that the beliefs that a mother’s career has a negative impact on the children and family life often leads to a lack of support or even opposition to a career of a women with children [2].

## Limitations

This study also has limitations. As it is a single center study additional research is needed to demonstrate generalizability of the findings in regard to other institutions and contexts. Although a substantial absolute number of students and lecturers participated in the survey, the relative response rate was only 13% for students and 11% for lecturers. This may have a potential effect on the study results due to the bias in the selection of the students and lecturers. However, our response rate is in the range of what is generally achieved with email-initiated surveys [47].

Furthermore, there might be a misinterpretation of having experienced discrimination and/or sexual harassment and having witnessed or observed it.

## Conclusion

Discrimination and sexual harassment at the workplace and in education institutions are global public health issues and do have detrimental effects on health, performance, work satisfaction, commitment to the workplace and thus productivity and innovation potential. They are prevalent in academic medicine among medical students, dental students and students of further study programs in the healthcare sector as well as lecturers. There are differences in the frequency, situations, perceived reasons and sources of discrimination and sexual harassment between students and lecturers and between the study programs. Female students and female faculty members are more often victims of discrimination and harassment.

Specific programs for lecturers and students are necessary to raise awareness, educate the faculty about discrimination and sexual harassment and elaborate plans on how to prevent and respond to it and whom to address. Requirements for such courses should be integrated into accreditation guidelines for study programs. Special attention should be paid to women.

A reporting tool should be implemented to monitor the frequency of discriminatory or undervaluing behavior and sexual harassment. Adequate actions should be introduced to follow up on the results of the monitoring. Further quantitative and qualitative studies are suggested to learn more about the specific situations and support needed. Already developed and implemented institutional guidelines should be evaluated and assessed.

National preventive strategies should be implemented to tackle issues of discrimination and harassment at the workplace and in higher education institutions for the different target groups.

### Supplementary Information

**Additional file 1.** Questionnaire.

## Data Availability

The datasets generated and/or analysed during the current study are not publicly available due to anonymous request of the survey and deliberate participation but are available from the corresponding author on reasonable request.
