# Supplementary material for: Perceptions of lecturers and students regarding discriminatory experiences and sexual harassment in academic medicine – results from a faculty-wide quantitative study
Source: BMC Med Educ. 2024 Apr 24;24:447. doi: 10.1186/s12909-024-05094-x (PMC11044556; doi:10.1186/s12909-024-05094-x)
Supplement: Supplementary file 1 — Additional file 1. Questionnaire. [file 12909_2024_5094_MOESM1_ESM.docx]

**Questionnaire**

**Part I Discriminatory Experiences**

Treating each other fairly and respectfully is not always a matter of course.

Please assess in retrospect whether you have witnessed and/or experienced any form of discrimination at the faculty.

1. Discrimination occurs at the faculty…

*very frequently / frequently / occasionally / rarely / not at all / do not know*

1. Did you experience and/or observe either discriminatory or undervaluing behavior at the faculty?

*yes, experienced / yes, observed / yes, experienced and observed / no*

1. How often did you experience and/or observe either discriminatory or undervaluing behavior at the faculty?

*once / several times (<10) / frequently (>10)*

1. I attribute the discriminatory experiences I have experienced or observed to the following categories (multiple answers are possible):

*sex, gender/ performance, skills / nationality / language / age / socio-economic background / political reasons / colour of skin / sexual identity / religion / parenthood / health status / care for relatives / other issues*

1. From which persons or groups of people did this discriminatory behavior emanate? (multiple answers are possible):

*lecturers / patients / students / colleagues / executives and heads of everyday student life / directors, supervisors / visitors / nursing staff / supervisors of term papers and dissertations, habilitation / administrative staff / others*

1. In which of the following situations have you experienced or observed discriminatory behavior?

*lectures, seminars / practical courses / work situations / on the campus ( e.g. in the cantine or library ) / during exams*

**Part II Sexual Harassment**

Sexual harassment is a particular form of transgression that is also a well-known problem in the academic context. Regarding the following questions, we understand sexual harassment as sexually oriented behavior that is unwelcome, manifests itself in verbal, non-verbal or physical form and violates a person's dignity.

In the following questions, we would like to ask you to assess whether and in what form you have experiences or observed sexual harassment during your time at the Charité.

1. Did you experience some form of sexual harassment during your time at the Charité, e.g. through salacious remarks, unwelcome advances, explicit sexual acts?

*yes, I have experienced sexual harassment / yes, I have observed sexual harassment / yes, I have experienced and observed sexual harassment*

1. During your time at the Charité did you experience the following? (multiple answers are possible)

*-someone has spoken derogatorily of women, men, homosexuals or other sexes*

*-someone has sent you or another person derogatory or obscene jokes and sayings or pornographic or nude pictures by telephone, letter, e-mail, SMS or social media*

*-someone has made lewd remarks about you or another person, your appearance, your clothing or sexual allusions or derogatory remarks*

*-someone has whistled at you or another person unwantedly, stared immorally or gotten undressed with glances*

*-someone has made intrusive sexual offers or unwanted invitations with sexual intentions*

*-someone has promised you advantages if you accept sexual advances, or threatened you with disadvantages if you don't.*

*-someone has made unwanted physical contact, through apparently accidental touching or physically unnecessary proximity*

*-someone has forced you or another person into sexual acts*

1. **How often did you experience or observe this behavior?**

Once / several times (<10) / frequently (>10)

1. **From which persons or groups of people did this behavior emanate? (multiple answers are possible):**

lecturers / patients / students / colleagues / executives and heads of everyday student life / directors, supervisors / visitors / nursing staff / supervisors of term papers and dissertations, habilitation / administrative staff / others

Thank you for taking the time to answer these questions. If psychological stress or similar has arisen as a result of this survey and you need support, please contact XXX
